# Randomized controlled trial of the effects of high intensity and low-to-moderate intensity exercise on physical fitness and fatigue in cancer survivors: results of the Resistance and Endurance exercise After ChemoTherapy (REACT) study

**DOI:** 10.1186/s12916-015-0513-2

**Published:** 2015-10-29

**Authors:** Caroline S. Kampshoff, Mai J. M. Chinapaw, Johannes Brug, Jos W. R. Twisk, Goof Schep, Marten R. Nijziel, Willem van Mechelen, Laurien M. Buffart

**Affiliations:** VU University Medical Center, Department of Public & Occupational Health, and the EMGO Institute for Health and Care Research, Amsterdam, The Netherlands; VU University Medical Center, Department of Epidemiology and Biostatistics and the EMGO Institute for Health and Care Research, De Boelelaan 1089a, 1081 HV Amsterdam, The Netherlands; Máxima Medical Center, Department of Sports Medicine, Veldhoven, The Netherlands; Máxima Oncology Center, Eindhoven and Veldhoven, The Netherlands

**Keywords:** Exercise, Fatigue, Neoplasms, Physical fitness, Quality of life

## Abstract

**Background:**

International evidence-based guidelines recommend physical exercise to form part of standard care for all cancer survivors. However, at present, the optimum exercise intensity is unclear. Therefore, we aimed to evaluate the effectiveness of a high intensity (HI) and low-to-moderate intensity (LMI) resistance and endurance exercise program compared with a wait list control (WLC) group on physical fitness and fatigue in a mixed group of cancer survivors who completed primary cancer treatment, including chemotherapy.

**Methods:**

Overall, 277 cancer survivors were randomized to 12 weeks of HI exercise (n = 91), LMI exercise (n = 95), or WLC (n = 91). Both interventions were identical with respect to exercise type, duration and frequency, and only differed in intensity. Measurements were performed at baseline (4–6 weeks after primary treatment) and post-intervention. The primary outcomes were cardiorespiratory fitness (peakVO_2_), muscle strength (grip strength and 30-second chair-stand test), and self-reported fatigue (Multidimensional Fatigue Inventory; MFI). Secondary outcomes included health-related quality of life, physical activity, daily functioning, body composition, mood, and sleep disturbances. Multilevel linear regression analyses were performed to estimate intervention effects using an intention-to-treat principle.

**Results:**

In the HI and LMI groups, 74 % and 70 % of the participants attended more than 80 % of the prescribed exercise sessions, respectively (*P* = 0.53). HI (β = 2.2; 95 % CI, 1.2–3.1) and LMI (β = 1.3; 95 % CI, 0.3–2.3) exercise showed significantly larger improvements in peakVO_2_ compared to WLC. Improvements in peakVO_2_ were larger for HI than LMI exercise (β = 0.9; 95 % CI, −0.1 to 1.9), but the difference was not statistically significant (*P* = 0.08). No intervention effects were found for grip strength and the 30-second chair-stand test. HI and LMI exercise significantly reduced general and physical fatigue and reduced activity (MFI subscales) compared to WLC, with no significant differences between both interventions. Finally, compared to WLC, we found benefits in global quality of life and anxiety after HI exercise, improved physical functioning after HI and LMI exercise, and less problems at work after LMI exercise.

**Conclusions:**

Shortly after completion of cancer treatment, both HI and LMI exercise were safe and effective. There may be a dose–response relationship between exercise intensity and peakVO_2_, favoring HI exercise. HI and LMI exercise were equally effective in reducing general and physical fatigue.

**Trial registration:**

This study was registered at the Netherlands Trial Register [NTR2153] on the 5th of January 2010.

## Background

Exercise during and after cancer treatment is safe and may increase physical fitness [1], reduce fatigue [2], and enhance the health-related quality of life (HRQoL) [3]. International evidence-based guidelines have endorsed these findings and recommend physical exercise to be part of standard care for all cancer survivors [4]. However, current exercise recommendations remain rather generic. Defining the optimal mode, frequency, volume, and intensity of exercise in cancer survivors may help to further improve the effectiveness of exercise programs [5].

The effects of different exercise modes and volumes in breast cancer survivors during chemotherapy have been previously evaluated in large randomized controlled trials (RCT) [6, 7]. Yet, only two [8, 9] relatively small RCTs have studied the effects of different exercise intensities in cancer survivors after completion of primary cancer treatment. Burnham et al. [8] compared moderate versus low intensity aerobic exercise in breast cancer survivors (n = 18) and reported that both exercise programs improved cardiorespiratory fitness, compared to usual care, with no differences in effects between the interventions [8]. Gibbs et al. [9] reported larger improvements in cardiorespiratory fitness in breast cancer survivors (n = 73) after high intensity (HI) resistance exercise compared to low intensity exercise and usual care. Both high and low intensity exercise significantly improved muscle strength and reduced general fatigue compared to usual care, but no significant differences between the interventions were found [9]. Due to the scarcity of studies and small sample sizes, more insight into the effects of different exercise intensities is warranted to bridge this gap in existing knowledge.

Herein, we report results of the Resistance and Endurance exercise After ChemoTherapy (REACT) study [10]. This is the largest RCT to date that has examined the effectiveness of a HI and a low-to-moderate intensity (LMI) resistance and endurance exercise program compared with a wait list control (WLC) group in cancer survivors who had completed primary cancer treatment with cardiorespiratory fitness, muscle strength, and fatigue as primary outcomes. We included HRQoL, physical activity, daily functioning, body composition, mood, and sleep disturbances as secondary outcomes.

## Methods

### Design

The REACT study was a RCT including three study arms: HI exercise, LMI exercise, and a WLC group. The study was approved by the Medical Ethics Committee of the VU University Medical Center (Amsterdam) and the local ethical boards of all participating hospitals, including Máxima Medical Center (Eindhoven and Veldhoven), Catharina Hospital (Eindhoven), Elkerliek Hospital (Helmond), St. Anna Hospital (Geldrop), VieCuri Medical Center (Venray and Venlo), Zuwe Hofpoort Hospital (Woerden), St. Antonius Hospital (Utrecht and Nieuwegein), Academic Medical Center (Amsterdam), and Erasmus MC University Medical Center (Rotterdam).

### Participants

Between 2011 and 2013, patients were recruited from nine Dutch hospitals. Patients aged ≥18 years with histologically confirmed breast, colon, ovarian, cervix or testis cancer, or lymphomas with no indication of recurrent or progressive disease, who had completed (adjuvant or neo-adjuvant) chemotherapy were invited to participate. Exclusion criteria were (1) not being able to perform basic activities of daily living, (2) cognitive disorders or severe emotional instability, (3) other serious diseases that might hamper patients’ capacity of carrying out HI exercise (e.g. severe heart failure), and (4) inability to understand the Dutch language. Written informed consent was obtained from all patients prior to participation.

### Randomization and blinding

After baseline assessments, participants were stratified by cancer type and hospital, and randomly assigned to one of the three study arms. An independent research assistant performed the randomization by using a table of random numbers generated from statistical software. Allocation sequence was concealed from the clinical and research staff. Following randomization, both HI and LMI groups commenced their 12-week exercise program. Participants from the WLC group were similarly randomly allocated to HI or LMI. However, they started exercising after the post-test assessment. Study outcomes of objective physical assessments were assessed by trained and blinded assessors and participants were instructed not to reveal their group allocation.

### Exercise interventions

Full details of the HI and LMI programs are described elsewhere [10]. Both interventions were identical with respect to exercise type, duration and frequency, and differed only in intensity (Table 1) [10]. After medical clearance by a sports physician, exercise sessions were given twice per week for 12 weeks under supervision of a physiotherapist.

**Table 1:** Exercise intensities of the high intensity (HI) and low-to-moderate intensity (LMI) resistance and endurance exercise programs

	Resistance exercises (1-RM) ^a^	Endurance interval exercises	Endurance interval exercises	Counseling
	(six exercises targeting the large muscle groups)	Part A (MSEC) ^a^ (8 min alternating workload)	Part B (HRR) ^a^ (3 × 5 min constant workload)	
HI exercise ^b^	70–85 %	30/65 %	≥80 %	Participants were encouraged to start or maintain a physically active lifestyle in addition to the supervised exercise sessions
LMI exercise ^b^	40–55 %	30/45 %	40–50 %	

1-RM, One-repetition maximum; MSEC, Maximum short exercise capacity; HRR, Heart rate reserve

^a^ Every 4 weeks (weeks 1, 5, and 9), the physiotherapist evaluated training progress and adjusted the workload accordingly

^b^ Exercises were accompanied with BORG scores and heart rate monitors to guide the physiotherapists. In cases where the training intensity seemed too high or too low, the 1-RM, MSEC, or HRR were reassessed

Both exercise programs included six resistance exercises targeting large muscle groups with a frequency of two sets of 10 repetitions. Workload per exercise was defined by an indirect one-repetition maximum (1-RM) measurement. HI resistance exercises started in the first week at 70 % of 1-RM (Table 1) and gradually increased to 85 % of 1-RM in week 12, whereas LMI resistance exercises started at 40 % of 1-RM gradually increased to 55 % of 1-RM. Every 4 weeks (weeks 5 and 9) the physiotherapist conducted the indirect 1-RM test and adjusted the workload accordingly.

Furthermore, both programs included two types of endurance interval exercises, aiming to maximize improvements in cardiorespiratory fitness. In the first 4 weeks, patients cycled 2 × 8 minutes with alternating workloads. Workloads were defined by the maximum short exercise capacity (MSEC) estimated by the steep ramp test [11]. The HI group cycled 30 seconds at a workload of 65 % of the MSEC and 60 seconds at 30 %, and the LMI group cycled 30 seconds at a workload of 45 % of the MSEC and 60 seconds at 30 %. Once the first 4 weeks were accomplished, the duration of the latter block was reduced from 60 to 30 seconds in both exercise programs. Every 4 weeks, the physiotherapist evaluated training progress by means of the steep ramp test, and the workload was adjusted accordingly.

From the fifth week onwards, one additional endurance interval session was performed in exchange for one block of 8 minutes cycling. This interval session consisted of 3 × 5 minutes cycling at constant workload, with 1 minute rest between each bout. Participants trained on ergometers (e.g. cycle ergometer or treadmill). The workload was defined by the heart rate reserve (HRR) using the Karvonen formula [12]. The HI group trained at ≥80 % of HRR and the LMI group at 40–50 % of HRR.

The physiotherapists closely monitored individual session attendance. In addition, they applied behavioral motivation counseling techniques to overcome possible exercise barriers and to encourage participants to start or maintain a physically active lifestyle outside the exercise program. Participants were stimulated to be physically active at moderate intensity for at least 30 minutes, three times per week complementary to the supervised exercise program and regardless of their group allocation. The combination of supervised exercise, twice a week, and home-based exercises, three times a week, meets the recommendations of the evidence-based physical activity guidelines for cancer survivors [4].

### Measurements

All outcome measures were assessed at baseline (4–6 weeks after completion of primary cancer treatment) and after 12 weeks. Details on the validity and reliability of the different outcome measures have been described previously [13].

#### Primary outcome measures

Cardiorespiratory fitness was measured during a maximal exercise test on an electronically braked cycle ergometer according to a ramp protocol, aiming to achieve peak oxygen uptake (peakVO_2_, in mL/kg/min) within 8–12 minutes [14]. Expired gases were collected and analyzed breath by breath to determine peakVO_2_ [14]. PeakVO_2_ was defined as the highest values of oxygen consumption averaged over a 15 seconds interval within the last minute of exercise. After each test, peakVO_2_, peak power output (in watt), and the ventilatory threshold determined by the oxygen equivalent method were recorded.

Upper body muscle strength was assessed using a JAMAR hand grip dynamometer [15]. Participants were instructed to complete three measurements for each hand while alternating sides. The mean score of the three attempts of a participants’ dominant hand was used as indicator for upper body muscle strength. Lower body function was assessed using the 30-second chair-stand test [16]. Participants were instructed to rise to a full stand and return to the original seated position as quickly as possible. The total number of times that the participant raised to a full stand in 30 seconds was reported. Both the hand grip strength and 30-second chair-stand tests are valid outcome measures and can be used to characterize upper body strength and lower body function [15, 17].

Fatigue was assessed using the Multidimensional Fatigue Inventory (MFI) questionnaire [18]. The MFI is a validated questionnaire and consists of 20 items divided into five subscales: general fatigue, physical fatigue, reduced physical activity, reduced motivation, and mental fatigue. Participants were asked to indicate, on a 1–5 scale, to what extent the particular item applied to them, with a maximum sum score of 20 points per subscale.

#### Secondary outcome measures

HRQoL was measured using the European Organisation Research and Treatment of Cancer-Quality of Life questionnaire-Core 30 [19], anxiety and depression by the Hospital Anxiety and Depression Scale [20], sleep disturbances with the Pittsburgh Sleep Quality Index [21], participation in daily life using the Impact on Participation and Autonomy (IPA) [22], and self-reported physical activity (PA) using the Physical Activity Scale for the Elderly questionnaire [23]. Objective measurement of PA was assessed with an accelerometer (Actitrainer, Actigraph, Fort Walton Beach, USA) using vertical accelerations converted into PA counts per minute. Participants were instructed to wear the accelerometer around the hips for seven consecutive days during all waking hours. Raw data was recorded in epochs of 60 seconds. A valid day of wearing-time was defined as 10 hours and non-wearing time was defined as 90 minutes of consecutive zero counts. Raw data were processed using ActiLife Software version 6.10.2 (ActiGraph, Pensacola, Florida, USA). Body weight was measured to the nearest 0.1 kg on a digital scale with light cloths on and no shoes. Body height was measured to the nearest 0.1 centimeters without shoes. Body mass index was calculated from the measured body weight and height accordingly. Thickness of four skinfolds in millimeters (biceps, triceps, suprailiac, and subscapular) was measured using a Harpenden skinfold caliper. The mean of two consecutive measurements was used for further analyses.

#### Assessments of covariates, session attendance, adverse events, and contamination

Sociodemographic data were collected by self-report. Clinical information was obtained from medical records. Physiotherapists monitored session attendance in the exercise logs, as well as possible adverse events during the intervention period. In addition, adverse events were documented from the medical records for the intervention and WLC groups. Contamination was assessed by asking participants from the WLC group at the post-test assessment if they had attended supervised exercise outside the study [24].

### Power calculations

Power calculations were based on a previous uncontrolled trial evaluating the effectiveness of a HI resistance and endurance exercise program in 119 cancer survivors after completion of chemotherapy [25]. To be able to detect a difference in peakVO_2_ of 3 mL/min/kg (SD = 5.8), with a power of 0.80 and two-sided alpha of 0.05, 60 participants per group were needed at post-test assessment. To compensate for loss to follow-up (20–40 %) and taking into account the multilevel design, a sample size of 280 was required. Additional power calculations (a power of 0.80 and alpha of 0.05) for fatigue (MFI) and hand grip strength demonstrated that this sample size of 280 would also be sufficient to detect a clinically relevant difference of two points [26] on the MFI questionnaire and a difference of 3 kg (10 % difference) in hand grip strength.

### Statistical analyses

Differences in age, sex, and diagnosis between participants and non-participants were examined using multivariable logistic regression analyses.

For all outcome measures, we used multivariable multilevel linear regression analyses to evaluate differences in effects between the HI, LMI, and WLC groups. Possible clustering of data within hospitals was taken into account using a two-level structure with hospital as the first level and participants as the second. Both interventions were simultaneously regressed on the post-test value of the outcome, adjusted for the baseline value, with age and sex as covariates. All analyses were performed according to an intention-to-treat principle. In addition, exploratory analyses were conducted to check for effect modification by age, sex, and diagnosis (breast cancer vs. other). To determine whether missing data were selective, univariable logistic regression analyses were conducted to examine baseline differences in the primary outcomes between participants who completed post-test assessments and those who did not (dropouts). We found no significant differences between the groups, and consequently, we considered missing values to be at random. Since also dropout rate was 10 %, we did not use imputation strategies [27]. We considered *P* <0.05 to be statistically significant. The statistical analyses were performed using MLwiN (version 2.22) and Statistical Package of Social Sciences (SPSS, version 20.0).

## Results

**Fig. 1:**
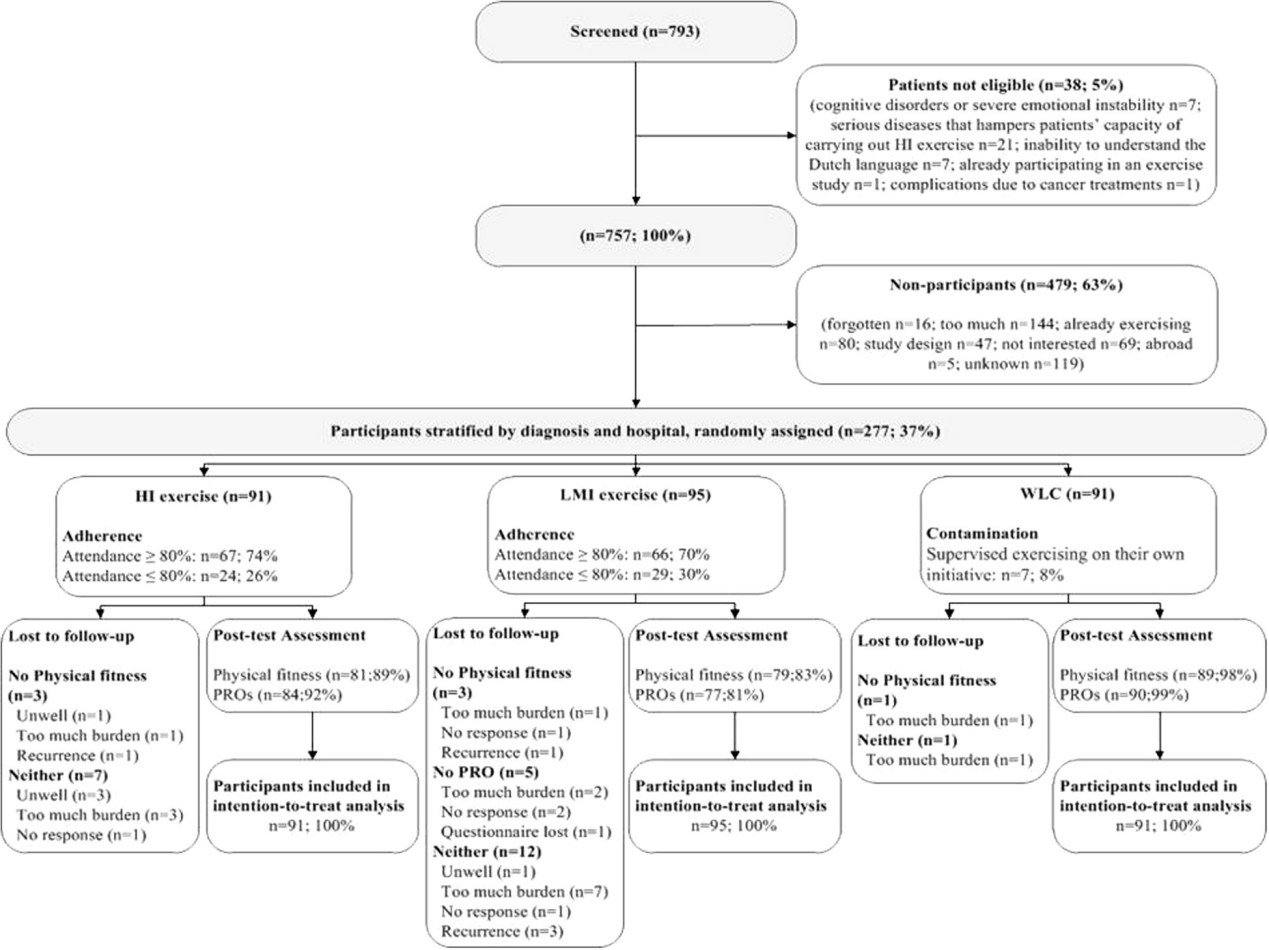
Patients flowchart of the REACT study. HI, High intensity exercise; LMI, Low-to-moderate intensity exercise; WLC, Wait list control group; PRO, Patient reported outcomes

Of the 757 patients who were eligible, 277 (37 %) participated (Fig. 1). No significant differences in age, sex, and diagnosis were found between the participants and non-participants (Table 2). Furthermore, sociodemographic and clinical data of the participants in the intervention and WLC groups were balanced at baseline (Table 2). In the HI and LMI groups, 74 % and 70 % of the participants attended more than 80 % of the prescribed exercise sessions, respectively (*P* = 0.53; Fig. 1).

**Table 2:** Baseline characteristics of participants and non-participants

Characteristics	HI	LMI	WLC	Non-participants
	n = 91	n = 95	n = 91	n = 479
Sociodemographic				
Age, mean (SD) years	54 (11.0)	53 (11.3)	54 (10.9)	55 (11.6)
Sex, n (%) male ^a^	18 (20)	17 (18)	20 (22)	77 (16)
Married/living together, n (%) yes	73 (80) ^f^	87 (92) ^f^	72 (79) ^f^	
Education, n (%) ^b^				
Low	19 (21)	12 (13)	16 (18)	
Intermediate	37 (41)	43 (46)	42 (46)	
High	34 (38)	38 (40)	33 (36)	
Being employed, n (%)				
Employed	54 (59)	56 (58)	57 (63)	
Not employed	25 (28)	22 (23)	19 (21)	
Retirement	12 (13)	17 (18)	15 (17)	
Smoking, n (%) yes ^c^	7 (8)	5 (5)	5 (6)	
Comorbidity, n (%) yes	12 (13)	8 (8)	10 (11)	
Sport history, n (%) yes ^d^	45 (50)	61 (65)	49 (54)	
Exercise during chemotherapy, n (%) yes ^b^	21 (23)	21 (22)	10 (11)	
Clinical				
Diagnosis, n (%) ^e^				
Breast	62 (68)	62 (65)	57 (63)	309 (65)
Colon	15 (17)	19 (20)	15 (17)	85 (18)
Ovarian	4 (4)	3 (3)	5 (6)	23 (5)
Lymphoma	9 (10)	9 (9)	8 (9)	47 (10)
Cervix	0 (0)	2 (2)	2 (2)	12 (3)
Testis	1 (1)	0 (0)	4 (4)	4 (1)
Stage of disease, n (%)				
Stage I–II	68 (75)	57 (60)	62 (68)	
Stage II–IV	23 (25)	38 (40)	29 (32)	
Type of treatment, n (%) yes				
Surgery	83 (91)	87 (92)	80 (88)	
Radiation therapy	46 (51)	41 (43)	48 (53)	
Surgery + radiation therapy	41 (45)	39 (41)	46 (51)	
Immunotherapy	16 (18)	25 (26)	18 (20)	
Hormonal therapy	45 (50)	40 (42)	43 (47)	
Type of chemotherapy, n (%)				
TAC	39 (43)	33 (34)	31 (34)	
FEC	7 (8)	7 (7)	5 (6)	
TAC/FEC combinations	15 (17)	21 (22)	17 (19)	
Capecitabine and oxaliplatin	8 (9)	11 (12)	7 (8)	
Oxaliplatin combinations	7 (8)	8 (8)	7 (8)	
Carboplatin and paclitaxel	4 (4)	4 (4)	10 (11)	
CHOP	5 (6)	6 (6)	7 (8)	
ABVD	4 (4)	2 (2)	2 (2)	
Cisplatin	0	2 (2)	1 (1)	
BEP	1 (1)	0	3 (3)	
Other	1 (1)	1 (1)	1 (1)	

HI, High intensity; LMI, Low-to-moderate intensity; WLC, Wait list control; FEC, Fluorouracil, epirubicin, cyclophosphamide; TAC, Taxotere, adriamycin, cyclophosphamide; CHOP, Cyclophosphamide, doxorubicin, vincristine, prednisone; ABVD, Doxorubicin, bleomycin, vinblastine, dacarbazine; BEP, Bleomycin, etoposide, cisplatin

^a^ n-4 (non-participants)

^b^ n-3

^c^ n-4

^d^ n-1

^e^ n-1 (non-participants)

^f^ *P* <0.05

### Exercise effects on primary outcomes

HI (β = 2.2; 95 % CI, 1.2–3.1) and LMI (β = 1.3; 95 % CI, 0.3–2.3) showed significantly larger improvements in peakVO_2_ compared to WLC (Table 3). Improvement in peakVO_2_ was larger for HI than LMI (β = 0.9; 95 % CI, −0.1 to 1.9), but the difference was not statistically significant (*P* = 0.08). Relative improvements in peakVO_2_ were 20 % and 15 % for HI and LMI, respectively, which is in line with the relative improvements in healthy adults after a 12-week exercise program [28]. No significant intervention effects were found for grip strength and 30-second chair-stand tests. Compared to WLC, both HI and LMI showed significant improvements in general fatigue (HI: β = −1.3; 95 % CI, −2.2 to −0.4 and LMI: β = −1.1; 95 % CI, −2.0 to −0.2), physical fatigue (HI: β = −2.0; 95 % CI, −2.9 to −1.1 and LMI: β = −1.4; 95 % CI, −2.3 to −0.5), and reduced activity (HI: β = −1.1; 95 % CI, −1.9 to −0.2 and LMI: β = −1.2; 95 % CI, −2.1 to −0.3), with no significant differences between both interventions. HI showed a beneficial effect on motivation compared to LMI (β = −0.8; 95 % CI, −1.5 to −0.03) and WLC (β = −1.2; 95 % CI, −1.9 to −0.4), with no significant differences between LMI and WLC. Furthermore, HI showed a significant reduction in mental fatigue compared to WLC (β = −0.9; 95 % CI, −1.7 to −0.2). The effects on peakVO_2_ were modified by age (HI: β_interaction_ = −0.2; 95 % CI, −0.3 to −0.1; *P* = 0.000 and LMI: β_interaction_ = −0.1; 95 % CI, −0.2 to −0.01; *P* = 0.03), indicating larger effects for younger participants. No significant interaction effects for gender or diagnosis were found for physical fitness or fatigue.

**Table 3:** Mean (SD) values of baseline and post-test measurement differences in effects on primary outcomes physical fitness and fatigue between groups ^a^

	High intensity (HI)		Low-to-moderate intensity (LMI)		Wait list control (WLC)		HI vs. WLC	LMI vs. WLC	HI vs. LMI
	Baseline mean (SD)	Post-test mean (SD)	Baseline mean (SD)	Post-test mean (SD)	Baseline mean (SD)	Post-test mean (SD)	β (95 % CI)	β (95 % CI)	β (95 % CI)
Primary outcomes									
Cardiorespiratory fitness ^b^									
PeakVO_2_ (mL/kg/min)	21.9 (6.5)	26.3 (7.6)	22.3 (5.9)	25.6 (6.5)	21.5 (5.5)	23.8 (5.9)	**2.2 (1.2 to 3.1)** ^h^	**1.3 (0.3 to 2.3)** ^h^	0.9 (−0.1 to 1.9) ^i^
Peak power output (W)	136 (46)	163 (53)	134 (43)	154 (45)	135 (42)	150 (43)	**12.6 (7.7 to 17.5)** ^h^	**5.0 (0.01 to 9.9)** ^h^	**7.6 (2.5 to 12.7)** ^h^
Ventilatory threshold (mL/kg/min)	15.6 (4.1)	18.8 (4.7)	16.2 (4.8)	18.8 (5.2)	15.5 (4.8)	17.3 (5.6)	**1.5 (0.4 to 2.5)** ^h^	**1.1 (0.1 to 2.2)** ^h^	0.4 (−0.7 to 1.4)
Muscle strength									
Sit to stand (stands) ^c^	17 (4.4)	19 (4.9)	16 (3.6)	19 (4.8)	16 (3.6)	18 (3.9)	0.2 (−0.8 to 1.1)	0.6 (−0.3 to 1.5)	−0.5 (−1.4 to 0.4)
Grip strength (kg) ^d^	32.5 (9.7)	34.4 (10.5)	32.9 (9.8)	34.9 (9.8)	33.5 (9.5)	35.5 (10.6)	−0.3 (−1.3 to 0.7)	0.3 (−0.7 to 1.3)	−0.6 (−1.6 to 0.5)
Fatigue (Range 4–20) ^e^									
General fatigue ^f^	12.8 (3.8)	10.0 (3.3)	12.6 (4.1)	10.1 (3.4)	12.7 (4.2)	11.3 (4.1)	**−1.3 (−2.2 to −0.4)** ^h^	**−1.1 (−2.0 to −0.2)** ^h^	−0.2 (−1.1 to 0.7)
Physical fatigue ^f^	12.8 (3.9)	9,0 (3.2)	12.3 (3.9)	9.4 (3.6)	13.2 (4.0)	11.2 (3.9)	**−2.0 (−2.9 to −1.1)** ^h^	**−1.4 (−2.3 to −0.5)** ^h^	−0.6 (−1.6 to 0.3)
Reduced activity ^g^	12.2 (3.8)	9.6 (3.2)	11.5 (3.6)	9.1 (3.5)	11.8 (3.6)	10.5 (3.6)	**−1.1 (−1.9 to −0.2)** ^h^	**−1.2 (−2.1 to −0.3)** ^h^	0.1 (−0.8 to 1.0)
Reduced motivation ^g^	9.4 (3.2)	7.9 (2.6)	9.0 (3.0)	8.5 (3.1)	8.6 (3.1)	8.7 (3.2)	**−1.2 (−1.9 to −0.4)** ^h^	−0.4 (−1.1 to 0.4)	**−0.8 (−1.5 to −0.03)** ^h^
Mental fatigue ^f^	11.1 (4.2)	9.8 (3.7)	10.9 (4.0)	9.9 (3.6)	10.7 (4.1)	10.5 (4.1)	**−0.9 (−1.7 to −0.2)** ^h^	−0.7 (−1.5 to 0.1) ^i^	−0.2 (−1.1 to 0.6)

^a^ Adjusted model, corrected for age and sex

^b^ Missing due to technical problems (n = 5), musculoskeletal problems (n = 1), or discomfort (n = 6)

^c^ Missing due to musculoskeletal problems (n = 2)

^d^ Missing due to technical problems (n = 3) or musculoskeletal problems (n = 2)

^e^ Higher score means a higher level of self-reported fatigue in all subscales

^f^ Missing due to incomplete questionnaire (n = 1)

^g^ Missing due to incomplete questionnaire (n = 2)

^h^ *P* <0.05)

^i^ 0.05 ≤ *P* <0.10

### Exercise effects on secondary outcomes

HI showed significantly larger improvements in global quality of life (QoL) (β = 5.9; 95 % CI, 2.0–9.8) and reduced anxiety (β = −1.0; 95 % CI, −1.7 to −0.3) compared to WLC (Table 4). Significantly larger improvements in physical functioning were found for both exercise programs compared to WLC (HI: β = 3.1; 95 % CI, 0.7–5.5 and LMI: β = 4.1; 95 % CI, 1.6–6.6), with no significant differences between the exercise programs. The effects of HI on global QoL were larger for younger participants (β_interaction_ = −0.4; 95 % CI, −0.8 to −0.04; *P* = 0.03) and for participants with breast cancer (β_interaction_ = 9.5; 95 % CI, 1.4–17.8; *P* = 0.02). Women showed larger improvements after HI in global QoL (β_interaction_ = 11.1; 95 % CI, 1.8–20.4; *P* = 0.02) and physical functioning (β_interaction_ = 7.1; 95 % CI, 1.2–13.0; *P* = 0.02) than men. No significant between-group differences were found for role, emotional, cognitive, and social functioning, body composition, sleep disturbances, physical activity levels, and depression, nor for the IPA questionnaire, except for significantly lower scores on the ‘problems at work’ subscale after LMI (β = −0.3; 95 % CI, −0.6 to −0.02) compared to WLC (Table 4, 14 subscales of IPA are not presented).

**Table 4:** Baseline and post-test measurements and adjusted between group differences on secondary outcomes of health-related quality of life, body composition, sleep disturbances, physical activity, and distress ^a^

	High intensity (HI)		Low-to-moderate intensity (LMI)		Wait list control (WLC)		HI vs. WLC	LMI vs. WLC	HI vs. LMI
	Baseline mean (SD)	Post-test mean (SD)	Baseline mean (SD)	Post-test mean (SD)	Baseline mean (SD)	Post-test mean (SD)	β (95 % CI)	β (95 % CI)	β (95 % CI)
Health-related quality of life (Range 0–100) ^b^									
Global quality of life ^c^	72.8 (15.3)	82.0 (13.6)	73.6 (17.2)	79.7 (16.1)	71.0 (16.5)	75.3 (15.4)	**5.9 (2.0 to 9.8)** ^m^	3.3 (−0.6 to 7.2)	2.6 (−1.4 to 6.6)
Physical functioning	82.0 (13.8)	88.1 (9.5)	83.0 (12.2)	89.6 (10.2)	80.2 (15.4)	84.1 (13.1)	**3.1 (0.7 to 5.5)** ^m^	**4.1 (1.6 to 6.6)** ^m^	−1.0 (−3.5 to 1.5)
Role functioning ^c^	73.7 (25.1)	82.5 (21.4)	69.2 (25.8)	83.5 (21.1)	67.4 (25.6)	82.0 (21.4)	−2.1 (−7.6 to 3.3)	0.8 (−4.7 to 6.3)	−3.0 (−8.6 to 2.7)
Emotional functioning ^c^	86.0 (15.9)	88.3 (15.3)	82.9 (16.3)	84.0 (17.3)	83.5 (17.0)	83.3 (17.5)	3.3 (−0.4 to 7.1)	1.1 (−2.7 to 4.9)	2.2 (−1.7 to 6.1)
Cognitive functioning ^c^	79.9 (22.5)	84.3 (17.5)	78.0 (21.4)	80.3 (19.5)	76.7 (22.7)	80.6 (21.1)	2.0 (−2.4 to 6.3)	−0.9 (−5.3 to 3.6)	2.8 (−1.7 to 7.3)
Social functioning ^c^	78.3 (22.0)	89,6 (15.1)	78.2 (20.0)	86.1 (20.0)	75.6 (24.6)	85.2 (21.7)	3.1 (−1.7 to 7.9)	−0.2 (−5.1 to 4.7)	3.3 (−1.7 to 8.3)
Body composition									
Body mass index, kg/m^2^	26.8 (4.0)	26.8 (4.0)	26.3 (4.3)	26.5 (4.4)	27.7 (4.8)	27.7 (4.8)	−0.001 (−0.3 to 0.3)	0.2 (−0.1 to 0.4)	−0.2 (−0.4 to 0.1)
Sum of skinfolds, mm ^d^	73.0 (20.7)	72.2 (22.7)	72.2 (30.1)	73.1 (29.6)	77.7 (32.0)	78.2 (31.6)	−1.7 (−5.3 to 1.8)	−0.3 (−3.9 to 3.4)	−1.5 (−5.2 to 2.2)
Sleep disturbances (Range 0–21) ^e,f^									
	10.3 (3.3)	9.9 (3.3)	10.9 (3.1)	10.7 (3.7)	10.1 (3.2)	9.9 (3.6)	−0.2 (−1.0 to 0.6)	0.1 (−0.8 to 0.9)	−0.2 (−1.1 to 0.6)
Physical activity									
Self-reported PA ^g^	93.0 (71.5)	117.6 (87.7)	106.1 (80.9)	129.0 (68.5)	96.2 (66.2)	121.2 (72.9)	−2.0 (−20.7 to 16.7)	1.4 (−17.7 to 20.4)	−3.4 (−22.8 to 16.0)
Accelerometer, counts per minute ^h,i^	246 (95.8)	248 (106.3)	229 (91.0)	247 (76.6)	239 (89.5)	258 (87.0)	−12.9 (40.3 to 14.5)	−5.7 (−33.0 to 21.5)	−7.2 (−35.4 to 21.0)
Distress (Range 0–21) ^j^									
Anxiety ^k^	4.0 (3.0)	3.2 (2.9)	4.0 (3.0)	3.9 (3.3)	3.8 (2.8)	4.1 (3.0)	**−1.0 (−1.7 to −0.3)** ^m^	−0.4 (−1.0 to 0.3)	−0.6 (−1.3 to 0.1) ^n^
Depression ^l^	3.1 (2.7)	2.5 (2.6)	3.5 (3.2)	2.7 (2.8)	3.3 (2.8)	3.0 (3.2)	−0.4 (−1.1 to 0.2)	−0.4 (−1.1 to 0.2)	−0.02 (−0.7 to 0.6)

^a^ Adjusted model, corrected for age and sex

^b^ Higher score means a higher level of self-reported HRQoL in all subscales

^c^ Missing due to incomplete questionnaire (n = 1)

^d^ Missing due to skin problems (n = 3)

^e^ Higher score means poorer self-reported sleep quality

^f^ Missing due to incomplete questionnaire (n = 58)

^g^ Missing due to incomplete questionnaire (n = 1)

^h^ Average counts for Y-axis

^I^ Missing due to technical problems/insufficient wearing-time (n = 85)

^j^ Higher score means a higher level of anxiety and depression in both subscales

^k^ Missing due to incomplete questionnaire (n = 2)

^l^ Missing due to incomplete questionnaire (n = 3)

^m^ *P* <0.05

^n^ 0.05 ≤ *P* <0.10

### Adverse events

No adverse events directly related to the exercise programs were reported. Nevertheless, five participants reported disease recurrence and withdrew from the study, four participants withdrew from the study because of comorbidities not related to the interventions (i.e. heart failure, hernia nuclei pulposi, ankle fracture, and abdominal adhesions), six participants withdrew from the study because two exercise sessions per week was too much, and 11 participants reported musculoskeletal problems at the start of the exercise program and they continued with a modified program (despite program modifications, four of these participants withdrew).

## Discussion

We performed a head-to-head comparison of a 12-week HI and LMI exercise program compared to WLC shortly after completion of primary cancer treatment in a large group of cancer survivors with mixed diagnoses. This allowed us to determine differences in effects of exercise intensity on physical fitness, fatigue, and HRQoL.

Both HI and LMI significantly improved peakVO_2_ compared to WLC. We found mean peakVO_2_ improvements of 4.4 mL/kg/min after HI and 3.3 mL/kg/min after LMI, which is in line with the 3.3 mL/kg/min increase reported in a meta-analysis of three RCTs among patients who completed cancer treatment [1]. Improvements in peakVO_2_ tended to be larger after HI than LMI, suggesting a dose–response relationship for exercise intensity. However, this should be confirmed in future studies. Improving peakVO_2_ of cancer survivors is particularly important because, compared to healthy adults, their peakVO_2_ levels are very poor [29]. Higher peakVO_2_ levels in cancer survivors have been associated with lower fatigue and higher HRQoL [25, 30]. In addition, results from observational studies showed a positive association between peakVO_2_ and survival [31], but causality needs to be established.

In contrast to a meta-analysis examining effects of resistance exercises on muscle strength [32], we found no significant intervention effects on the grip strength and 30-second chair-stand tests. However, the indirect 1-RM tests that were conducted every 4 weeks as part of the exercise programs indicated an improvement of 37 % on the leg press and 34 % on the vertical row. Therefore, the current lack of intervention effects may be related to our choice of outcome measures. Although grip strength is a reliable and valid measure of general upper body muscle strength [15], it may not be sensitive enough to detect improvements in muscle strength of the upper arm and shoulder [33]. Comparably, the 30-second chair-stand test is a reliable and valid functional test [16], but may be prone to ceiling effects [34]. More direct measures of muscle strength, such as isokinetic dynamometers, may be more sensitive to detect changes over time [35].

Compared to WLC, both HI and LMI resulted in significant and clinically meaningful reductions in general fatigue, physical fatigue, and reduced activity. These results support previous results of a meta-analysis [2], showing that exercise significantly reduced cancer-related fatigue compared to non-exercise control groups. Interestingly, our results showed that exercise is beneficial in reducing both general and physical components of fatigue, regardless of the training intensity. From a physiological point of view, it is most likely that exercise counteracts physical fatigue [36]. Yet, HI also significantly reduced mental components of fatigue, compared to WLC. However, the intervention effects on reduced motivation and mental fatigue were small and may not be clinically relevant. Further research is needed to investigate whether combinations of exercise with cognitive behavioral therapy, stress management, or sleep therapy may have larger benefits on mental fatigue [37].

HI showed a significant and clinically meaningful (>10 points) increase on global QoL compared to WLC. Furthermore, HI and LMI significantly improved self-reported physical functioning. However, the improvements (6 points) may have small clinical meaning [38]. Both findings support the positive effect on global QoL and inconsistent findings on physical functioning reported in a previous meta-analysis [3]. A better understanding of the mechanisms underlying the exercise effects on HRQoL in cancer survivors may help to further target exercise interventions to specific HRQoL outcomes [39].

Previous meta-analyses reported small significant reductions in depression [40] and anxiety [3] after exercise. Our study supports these findings for anxiety, but not for depression. It has been suggested that larger effects on depression may only be expected in cancer survivors with higher levels of depression [40]. Our baseline data showed low mean values on the Hospital Anxiety and Depression Scale for depression and anxiety, leaving little room for improvement. Likewise, not many of our participants reported sleep disturbances or problems in daily functioning at baseline. Furthermore, we found no increase in PA levels in both exercise groups. However, our interventions included only a small component of behavioral motivation counseling and accomplishing behavior change may require more specific PA promotion strategies [41]. Finally, the lack of significant reductions in body fat was consistent with previous research [42] and not unanticipated, because both interventions focused on physical exercise only and did not aim at losing body weight by including a dietary component.

Strengths of this study include the direct comparison between HI and LMI, a well-designed (e.g. blinded outcome assessment, concealed allocation) multicenter RCT including a large sample size, the use of valid and reliable outcome measures, and intention-to-treat analyses. However, some limitations should be noted. First, compared to WLC, the reported effect sizes could be interpreted as modest. Nevertheless, the results from the current study highlight that twice-a-week, supervised exercise for 12 weeks is superior to natural recovery. Since adherence rates might have played a role in the magnitude of the effect sizes, a full report on adherence and compliance rates is needed to provide further insight on whether and how exercise components were delivered. Furthermore, participants of WLC were asked to maintain their habitual daily PA pattern; however, 8 % of the WLC participants engaged in weekly supervised exercise sessions and this may have reduced the intervention effects as well. Secondly, although we recruited 277 patients, 65 % of the participants were diagnosed with breast cancer and only small groups of other diagnosis were included. Therefore, potential differences in effects across different cancer types could not be established. Yet, except for global QoL, we found no differences in intervention effects between survivors of breast cancer or other types of cancer. Finally, slightly higher dropout rates were observed in LMI compared to the other groups. This can be partly explained by higher recurrence of disease rates in LMI, which was most likely coincidental.

## Conclusion

In conclusion, the current study demonstrates that supervised HI can be safely recommended to cancer survivors shortly after completion of cancer treatment. Because we found some indication for a dose–response relationship for peakVO_2_, HI may be preferred to LMI when aiming to improve peakVO_2_ levels in cancer survivors. Yet , HI and LMI were equally beneficial in counteracting general and physical fatigue. Additional research should further disentangle the effects of different exercise modes, frequencies, volumes, and intensities among different subgroups of patients to optimize evidence-based exercise recommendations for cancer survivors.

## Abbreviations

1-RM: One-repetition maximum
HI: High intensity
HRQoL: Health-related quality of life
HRR: Heart rate reserve
IPA: Impact on Participation and Autonomy
LMI: Low-to-moderate intensity
MFI: Multidimensional Fatigue Inventory
MSEC: Maximum short exercise capacity
PA: Physical activity
peakVO_2_: Peak oxygen uptake
QoL: Quality of life
RCT: Randomized controlled trial
WLC: Wait list control
